# Current and novel dual orexin receptor antagonists for the treatment of insomnia: the emergence of vornorexant

**DOI:** 10.1093/ijnp/pyaf077

**Published:** 2025-11-22

**Authors:** Yukihiro Chino, Hiroyuki Sugiyama, Shigeyuki Chaki, Yuri Sato

**Affiliations:** Medical Information, Taisho Pharmaceutical Co., Ltd., Toshima-ku, Tokyo, Japan; Medical Information, Taisho Pharmaceutical Co., Ltd., Toshima-ku, Tokyo, Japan; Medical Information, Taisho Pharmaceutical Co., Ltd., Toshima-ku, Tokyo, Japan; Research Headquarters, Taisho Pharmaceutical Co., Ltd., Kita-ku, Saitama, Japan; Medical Information, Taisho Pharmaceutical Co., Ltd., Toshima-ku, Tokyo, Japan

**Keywords:** DORA, insomnia, orexin receptor antagonist, vornorexant

## Abstract

Insomnia is a serious public health concern. As the widely prescribed hypnotics that positively modulate gamma-aminobutyric acid (GABA)_A_ receptor activity have various safety concerns, there is a growing demand for the development of novel hypnotics that act on targets other than GABA_A_ receptor signaling to overcome the drawbacks of current medications. As an alternative target to generate novel hypnotics, the orexin system has recently gained much attention, and 3 dual orexin receptor antagonists (DORAs)—suvorexant, lemborexant, and daridorexant—were launched. However, some doses of these DORAs are associated with a higher incidence of somnolence compared to placebo. Vornorexant is a novel DORA designed to have an improved pharmacokinetic profile—rapid absorption and the shortest half-life among existing DORAs to reduce the risk of residual activity, which has been confirmed in humans. Indeed, it has shown rapid sleep-promoting effects in patients with insomnia, maintaining its activity throughout the night but having a low incidence of next-day residual effects. In this review, we first provide an overview of the role of the orexin system in sleep/wake balance and then describe the profile of a newly developed DORA, vornorexant, from drug discovery to clinical results.

## INTRODUCTION: INSOMNIA AND CURRENT MEDICATIONS

According to population-based research, insomnia, a disorder characterized by difficulty in initiating or maintaining sleep and/or early morning awakening with an inability to resume sleep,^1^ affects approximately 30% of adults in various countries.^2^ Insomnia causes daytime hypersomnolence, mood disturbances, impaired memory, and inattentiveness, leading to deterioration in daily functioning and performance.^3^ Thus, insomnia is a serious public health concern and is associated with substantial individual and societal burden.

While cognitive behavioral therapy is the recommended first-line treatment, practical limitations such as resource scarcity, lack of adherence, and lack of efficacy for certain patients hinder its widespread adoption.^4^ Therefore, pharmacotherapy plays an important role in the treatment of insomnia. Currently, numerous effective pharmaceutical remedies are available for insomnia, most of which act on gamma-aminobutyric acid (GABA)_A_ receptor signaling, including benzodiazepines and Z-drugs.^5^ Although these drugs are widely and effectively used, their usage, particularly in the long term, is problematic owing to several drawbacks such as tolerance,^6^ cognitive decline and amnesia,^7^ risk of road traffic accidents,^8^ risk of falls,^9^ and dependence.^10^ These shortcomings have highlighted the need for alternative treatment options that promote sleep via a different mechanism of action. The orexin system has recently gained considerable attention as an alternative target for the generation of novel hypnotics.

## OREXIN SYSTEMS AND OREXIN RECEPTOR ANTAGONISTS

The orexin (also known as hypocretin) signaling system was discovered by 2 groups in 1998.^11^^,^^12^ Orexin peptides, orexin-A (hypocretin-1) and orexin-B (hypocretin-2), are derived from the common precursor prepro-orexin, which is synthesized mainly in the lateral hypothalamus. Orexin neurons send widespread projections to the brain, particularly dense projections to monoaminergic and cholinergic nuclei in the brain stem, where 2 subtypes of orexin receptors, orexin type 1 (OX_1_) receptor and orexin type 2 (OX_2_) receptor, are differentially expressed.^13^ Through these receptors, the orexin neurons exert a wide range of physiological events, including feeding,^12^ regulation of sleep–wake state,^14^ energy homeostasis,^15^ reward and addiction,^16^ and stress responses.^17^

The role of orexin in arousal was originally suggested by the findings that orexin deficiency and mutations in the gene encoding the OX_2_ receptor are responsible for the sleep disorder narcolepsy,^14^^,^^18^ which led to the hypothesis that the orexin system is necessary to maintain and stabilize wakefulness. Regulation of wake-controlling nuclei by orexin neurons, including the noradrenergic locus coeruleus, serotonergic dorsal raphe nucleus, cholinergic laterodorsal/pedunculopontine tegmental nuclei, and histaminergic tuberomammillary nucleus, all of which contain neurons expressing OX_1_ and/or OX_2_ receptors, may be responsible for the regulation of the arousal state and rapid eye movement (REM) sleep by orexin.^19^ Based on studies using genetically manipulated animals and ligands selective for each receptor, the OX_2_ receptor has been proposed to have an important role in the regulation of the arousal state, REM sleep, and non-REM sleep, while the OX_1_ receptor is thought to have additional effects on sleep–wake regulation, particularly the regulation of REM sleep. ^13^^,^^20^^,^^21^ Therefore, blockade of both orexin receptor subtypes was considered to have more potent effects than selective inhibition of each receptor, which led to the development of dual orexin receptor antagonists (DORAs) as a new class of hypnotics.

To date, several DORAs have been studied. The sleep-promoting effect of DORAs was demonstrated for the first time by using almorexant in rats, dogs, and healthy humans.^22^ Subsequently, almorexant was proven to be effective for patients with primary insomnia, demonstrating dose-dependent effects on sleep efficiency, objective latency to persistent sleep (LPS), and wake time after sleep onset (WASO)^23^ and dose-related improvements in total sleep time and WASO in elderly patients with primary insomnia.^24^ Despite these initial encouraging results, the development of almorexant was discontinued due to an unfavorable tolerability profile.^25^

Three DORAs have been launched for the treatment of insomnia in humans. The in vitro activities of DORAs at both the OX_1_ and OX_2_ receptors are summarized in Table 1. Suvorexant (Belsomra) was the first DORA^26^ to receive approval for primary insomnia; it was approved in 2014 in the United States of America (USA) and then in Japan, Canada, and Australia. Considering the presence of residual morning sleepiness presumably ascribed to the long elimination half-life (t_1/2_) of the drug (12.5 hours), the daily dose was reduced from 40 mg to 20 mg. Lemborexant (Dayvigo), a DORA^27^ with an effective t_1/2_ of 16.7 hours, has a maximum recommended dose of 10 mg. In contrast to other DORAs, lemborexant preferentially antagonizes the OX_2_ receptor versus the OX_1_ receptor.^26^ It was approved in 2019 in the USA and also in Japan, Canada, Australia, and other Asian countries. Daridorexant (Quviviq) is a DORA^28^ that was approved in 2022 in the USA and Europe and then in Canada and Japan, etc. It has a t_1/2_ of 5.9 hours, which is expected to minimize the residual effects. DORAs have been demonstrated to decrease wakefulness and increase both REM and non-REM sleep phases. This feature is quite distinct from that of benzodiazepines and Z-drugs, where alterations of the normal sleep architecture are observed.^29^ Moreover, all 3 approved DORAs have demonstrated sustained efficacy in long-term treatment and are devoid of safety risks, which are a concern with the long-term usage of benzodiazepines and Z-drugs; in particular, no evidence of physical dependence, tolerance, or rebound has been reported for DORAs during the treatment or upon treatment discontinuation,^30-34^ rendering them a viable option for longer-term treatment. Collectively, DORAs may provide a novel approach for treating insomnia without the problematic side effects observed with the use of drugs acting on GABA_A_ receptor signaling, such as benzodiazepines and Z-drugs.

**Table 1:** Structures and in vitro activities of DORAs.

Drug	Suvorexant	Lemborexant	Daridorexant	Vornorexant
*Chemical structure*	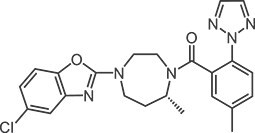	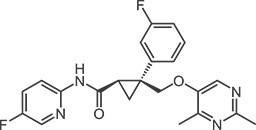	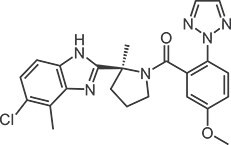	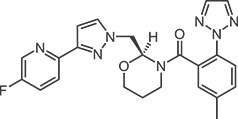
*Affinity (K_i_; nM)*				
Human OX_1_ receptor	1.23	9.99	8.8^a^	0.460
Human OX_2_ receptor	0.752	0.665	8.9^a^	0.374
Rat OX_1_ receptor	1.10	13.8		0.378
Rat OX_2_ receptor	0.903	0.739		0.566
*Antagonist activity (IC_50_; nM)*				
Human OX_1_ receptor	2.06	12.5	0.52^b^	1.61
Human OX_2_ receptor	4.81	1.26	0.78^b^	1.76

Values for suvorexant, lemborexant, and vornorexant are cited from Hikichi et al. (2025).^35^ Values for daridorexant are cited from Rappas et al. (2020)^36^ for affinity and Treiber et al. (2017)^37^ for antagonist activity.

a pK_i_ value

b K_b_ value.

Abbreviations: DORA, dual orexin receptor antagonist; IC_50_, 50% inhibitory concentration; K_b_, binding constant; K_i_, inhibition constant; OX_1_ receptor, orexin type 1 receptor; OX_2_ receptor, orexin type 2 receptor; p, predicted.

Although DORAs have demonstrated safety in clinical settings,^38^^,^^39^ some doses of these medications are associated with a higher incidence of somnolence. Several factors contribute to this effect, including the underlying insomnia disorder itself. Notably, the incidence of somnolence does not always correlate with exposure duration or t_1/2_; for example, a recent network meta-analysis reported that 25 mg, but not 50 mg, of daridorexant increased the risk of somnolence and 5 mg lemborexant which has a long t_1/2_, was not associated with somnolence.^40^ Nevertheless, the carry-over effect of medication remains one of the primary causes of next-day residual symptoms, and agents with shorter t_1/2_ may help reduce this risk. In this context, there is an urgent need to develop a DORA with the shortest t_1/2_ among existing agents to minimize the risk of next-day residual effects. Vornorexant (development code: TS-142) was developed as a new DORA (Table 1) with an improved PK profile for the treatment of insomnia—rapid absorption and brain penetration to exert rapid sleep onset and a t_1/2_ to maintain sleep-promoting effects through major parts of the night without next-day residual effects. Vornorexant was approved by the Pharmaceuticals and Medical Devices Agency in Japan for the treatment of insomnia in August 2025. In the following sections, we will review the drug discovery and preclinical and clinical profiles of vornorexant as well as its significance in the treatment of insomnia.

For reference, in addition to DORAs, seltorexant (JNJ-42847922), a selective OX_2_ receptor antagonist, has demonstrated sleep-promoting effects in a phase 2 study involving patients with insomnia.^41^ In this randomized clinical trial, seltorexant showed dose-dependent improvements in LPS and WASO over the first six hours, suggesting that selective OX_2_ receptor antagonists may also hold therapeutic potential for insomnia among drugs targeting the orexinergic system.

## DRUG DISCOVERY OF VORNOREXANT

Concerns about potential next-morning residual effects led to the goal of developing a new DORA that would work well as quickly as possible after administration and would reliably wear off the next morning. In the discovery of central nervous system (CNS) agents, shortening the t_1/2_ of a compound is more difficult than extending it, because the compound must cross the blood–brain barrier (BBB) to be delivered into the brain; additionally, the compound should have a high lipophilicity to penetrate BBB. Increasing a compound’s lipophilicity leads to a wider distribution within the body, resulting in a longer t_1/2_. However, to shorten the t_1/2_ of a compound, its lipophilicity must be reduced, thereby decreasing the amount distributed within the body (volume of distribution). This makes the compound less likely to cross the BBB. Efforts were made to reduce the compound’s lipophilicity to the bare minimum while maintaining its brain penetration.

**Figure 1:**
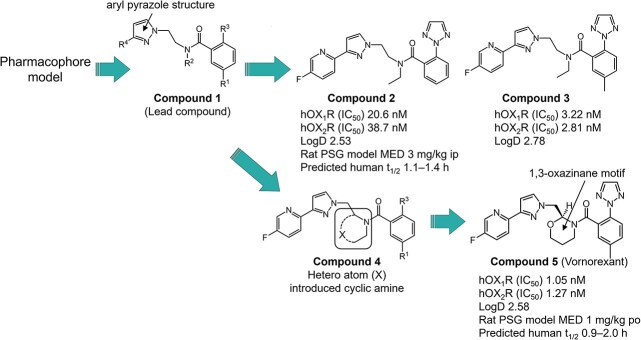
Structural modification of lead compounds to vornorexant resulted in improved pharmacological activity and pharmacokinetic profiles. hOX_1_R, human orexin type 1 receptor; hOX_2_R, human orexin type 2 receptor; HPLC, high-performance liquid chromatography; IC_50_, 50% inhibitory concentration; ip, intraperitoneal injection; log D, distribution coefficient at pH 7.4 measured by HPLC method; MED, minimum effective dose; PSG, polysomnography; t_1/2_, terminal half-life.

Initial investigations revealed that reducing a compound’s lipophilicity weakened its activity at the orexin receptor. Thus, we attempted to superimpose representative OX_1_ and OX_2_ receptor antagonists and observed that these molecules overlapped closely. Based on the superposition of these molecules, a novel pharmacophore model was created. A pharmacophore model is a three-dimensional model that represents the properties of the functional groups (hydrophobicity, hydrogen bond acceptor, hydrogen bond donor, and aromaticity) that are important for binding to target molecules, in this case, the orexin receptors. The pharmacophore model suggested that the cyclic amide linkages present in the representative antagonists could be replaced with a linear amide linkage.^42^ The design of the novel pharmacophore model and its fitting trials led to the creation of a pyrazoylethylbenzamide compound **1** as a linear linker. This was used as a lead compound for chemical modifications (Figure 1). The key component of the lead compound is the aryl pyrazole structure on the left side of the molecule. This partial structure ensures a balance between low lipophilicity and potent dual OX_1_ and OX_2_ receptor antagonist activities while providing the compound with good brain penetration.

The structure–activity relationship characterization of this novel class of DORAs led to the identification of compound **2** with modest in vitro antagonist activities for OX_1_ and OX_2_ receptors and an appropriate PK profile based on animal PK data (Figure 1). However, in a rat sleep study using polysomnography (PSG), compound **2** showed a marginal in vivo efficacy, which was consistent with its moderate in vitro potency. Compound **3** was identified as the most potent DORA among this class; unfortunately, it also had a slightly higher lipophilicity.^42^

To improve the activity while maintaining a low lipophilicity, a modification of the central portion of the molecule was deemed necessary. To maintain an active conformation, cyclization of the central core (as in SB-649868, a DORA) was considered based on the pharmacophore model. Conformational analysis revealed that the pyridyl-pyrazolyl-methyl side chain attached to the piperidine ring is likely to contribute to the compound’s active conformation. A simple cyclic amine, such as pyrrolidine or piperidine, showed potent antagonist activity, with single-digit, nano-molar potencies, but also increased the lipophilicity. A carbon atom on the pyrrolidine or piperidine core ring was replaced with a heteroatom such as an oxygen or sulfur atom (compound **4** in Figure 1) to reduce lipophilicity.^43^^,^^44^ Unfortunately, most of the cyclic compounds with an oxygen or sulfur atom in the 5- or 6-membered rings did not achieve both the highly potent antagonist activity and lower lipophilicity. However, compound **5**, possessing a unique 1,3-oxazinane motif, maintained antagonist activities for both OX_1_ and OX_2_ receptors and was about 20- to 30-fold more potent than compound **2** (Figure 1).^45^ Interestingly, compound **5** retained the highly potent antagonist activities despite having a lower lipophilicity compared with the parent piperidine. Compound **5** exhibited potent sleep-promoting effects at oral doses starting from 1 mg/kg in a rat PSG study and optimal PK properties with a rapid time to the maximum plasma concentration (C_max_; t_max_) and short t_1/2_ in rats and dogs, indicating a predicted human t_1/2_ of 0.9–2.0 hours. Based on its preclinical profile as a useful hypnotic with a low risk of next-day drowsiness, compound **5** (vornorexant) was selected as a candidate for further development.

## PRECLINICAL PHARMACOLOGICAL PROFILES OF VORNOREXANT

Vornorexant has high affinities for both human OX_1_ and OX_2_ receptors, with inhibition constants (K_i_ values) of 0.460 and 0.374 nM, respectively, and has shown potent antagonist activities at both receptors, with half-maximal inhibitory concentrations (IC_50_ values) of 1.61 and 1.76 nM, respectively (Table 1).^35^ In contrast, it did not show any appreciable affinities for a panel of 90 other receptors, transporters, and ion channels. Thus, vornorexant is a potent and selective DORA. The in vitro potency of vornorexant as a DORA is comparable to those of suvorexant and daridorexant,^37^ but differs from that of lemborexant exhibiting an approximately 10-fold greater potency against OX_2_ than OX_1_ receptors.^26^ Notably, vornorexant has been shown to inhibit radioligand bindings to both OX_1_ and OX_2_ receptors in a competitive manner, similar to other DORAs.^28^ Therefore, the binding mode of vornorexant may be similar to those of other DORAs. As predicted by the fact that the orexin system is well conserved across species,^19^ no species-specific differences have been observed in the in vitro activities of vornorexant (Table 1).

**Figure 2:**
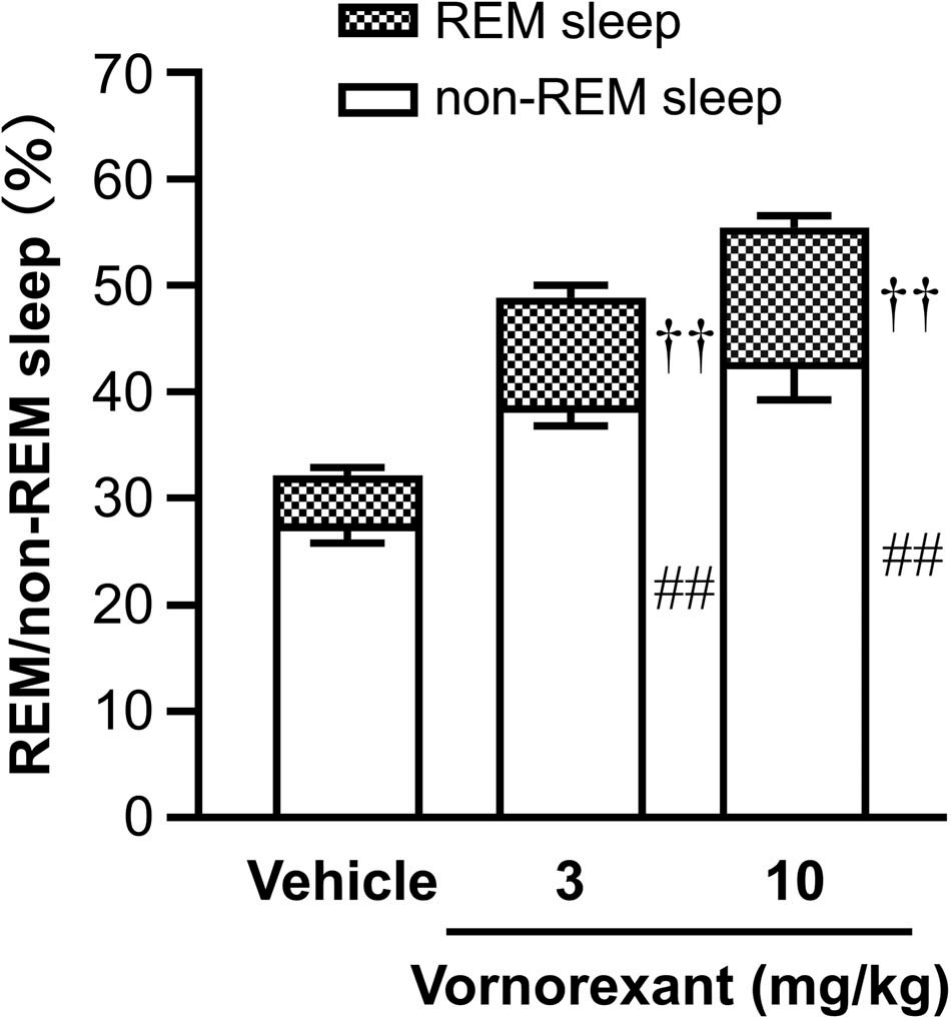
Effects of vornorexant on REM sleep and non-REM sleep in rats (adapted from Hikichi et al., 2025^35^). Mean percentage ± standard error (%) of REM and non-REM sleep during 2 hours after oral administration (*n* = 10–11). ^††^  *P*<.01 vs. control group for REM sleep; ^##^  *P*<.01 vs. control group for non-REM sleep (Dunnett’s test). REM, rapid eye movement.

**Figure 3:**
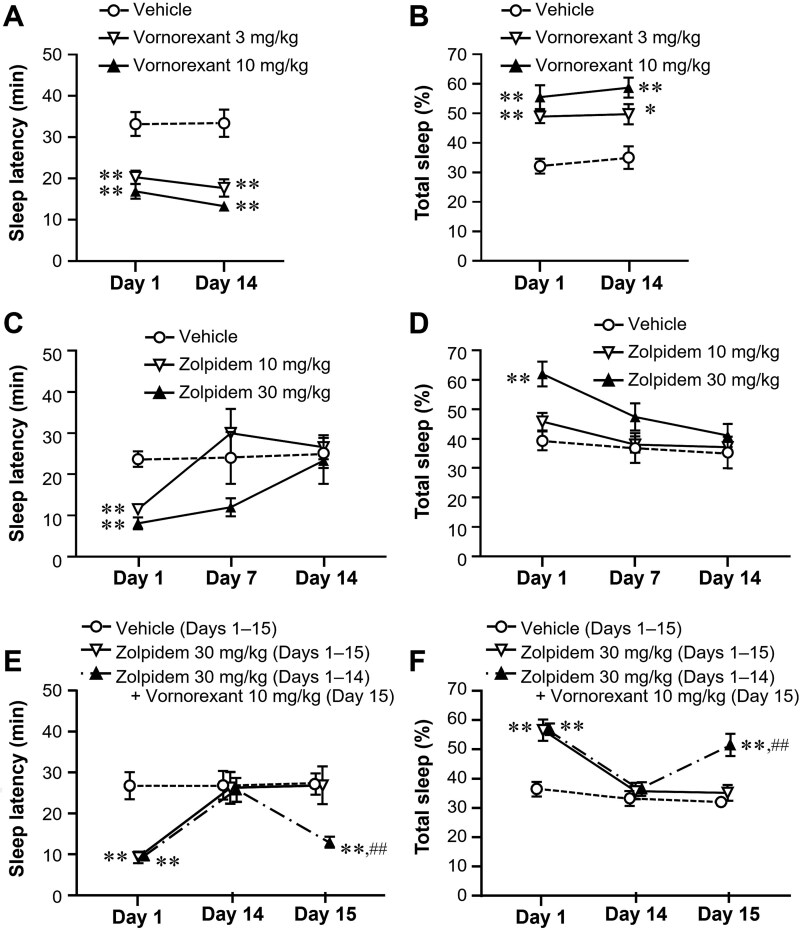
Effects of switching from subchronic zolpidem to vornorexant on sleep latency and total sleep duration within 2 hours after administration in rats (adapted from Hikichi et al., 2025^35^). Rats received once-daily dosing of vehicle, vornorexant (3 or 10 mg/kg, *n* = 10–11, A, B), or zolpidem (10 or 30 mg/kg, *n* = 7–8, C, D) for 14 days. Additionally, rats were dosed with zolpidem (30 mg/kg) for 14 days, followed by a single dose of vornorexant (10 mg/kg) on the 15th day. This group were compared with the vehicle group or zolpidem (30 mg/kg) group, which were dosed for 15 days (*n* = 10, E, F). Data are mean ± standard error. ^*^P<.05,^**^P<.01 compared with the vehicle group (Dunnett’s test). ^##^*P*<.01 compared with the zolpidem group (Student’s t-test).

Oral administration of vornorexant just before the start of the dark period (1, 3, or 10 mg/kg) resulted in a significant and dose-dependent decrease in sleep latency and increase in the percentage of time engaged in sleep in rats, indicating that vornorexant promotes sleep.^45^ Additionally, vornorexant increased both non-REM and REM sleep in rats (Figure 2),^35^ thus preserving the normal sleep architecture, in contrast to benzodiazepines or Z-drugs, which can alter the normal sleep architecture.^28^ Notably, vornorexant has shown sleep-promoting effects at doses at which the cerebrospinal fluid (CSF) drug concentration is just enough to exert pharmacological effects and occupy both OX_1_ and OX_2_ receptors.^35^ The sleep-promoting effect of vornorexant (3 and 10 mg/kg) was shown to be preserved after repeated treatment for 2 weeks (Figure 3A and B), while that of zolpidem, a Z-drug, was no longer observed (Figure 3C and D), showing that vornorexant does not cause the development of tolerance unlike benzodiazepines and Z-drugs. Interestingly, vornorexant exerted sleep-promoting effects in rats that developed tolerance after repeated treatment with zolpidem (Figure 3E and F). Thus, vornorexant may be effective in patients who have developed tolerance for current medications. Additionally, the combination of vornorexant and zolpidem demonstrated more potent sleep-promoting effects (both decrease in sleep latency and increase in total sleep time) than the individual drugs, indicating that vornorexant can be used concomitantly with Z-drugs to enhance their efficacy. Moreover, vornorexant can possibly reduce the side effects of Z-drugs by reducing the doses necessary to exert sleep-promoting effects. Notably, unlike zolpidem, vornorexant did not impair rotarod performance, even when used with ethanol.^35^

## PK PROFILE OF VORNOREXANT IN ANIMALS AND HEALTHY VOLUNTEERS

The absorption, distribution, metabolism, and excretion of ^14^C-labeled and unlabeled vornorexant have been investigated in vivo and in vitro, including in rat, dog, and human samples.^46^ The PK of vornorexant in humans was investigated in a double-blind study in healthy Japanese subjects, with a single ascending dose of 1–30 mg and multiple ascending doses of 10–30 mg.^47^ Vornorexant was rapidly absorbed in both animals and humans, with a t_max_ of 0.67 hours and 1.3 hours in rats and dogs, respectively, and 0.5–1.5 hours in humans after a single oral dosing under fasting conditions. The bioavailability of vornorexant in rats and dogs was observed to be 7.6% and 58.0%, respectively. No notable accumulation was observed with repeated administration in either animals or humans. Moreover, although food intake slightly delayed absorption, it did not change the C_max_, suggesting that the maximal effects of vornorexant may not be significantly affected. The extent of vornorexant metabolism varies between species, with the total body clearance being moderate in rats (2150 mL/h/kg) and low in dogs (163 mL/h/kg) and humans (apparent clearance, 10.5 L/h).

**Figure 4:**
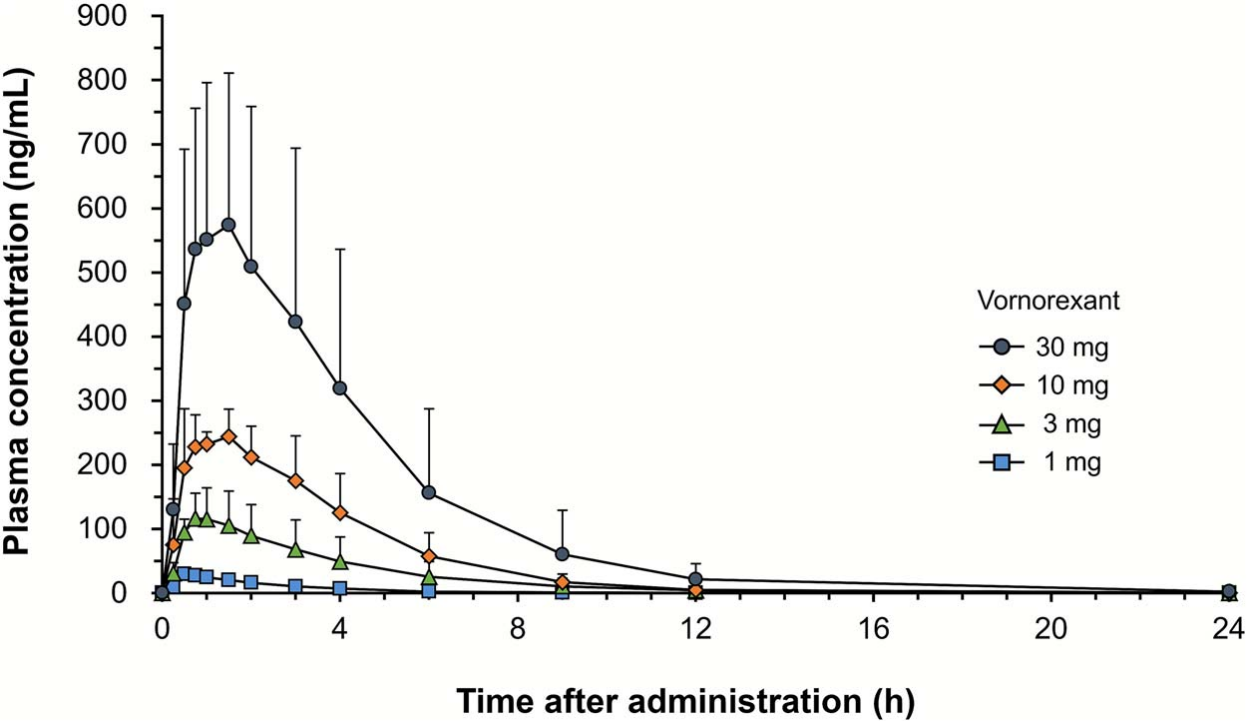
Plasma concentration–time profiles of vornorexant following a single oral dosing in healthy volunteers (adapted from Kambe et al., 2023^47^). Data are represented as the mean + standard deviation (*n* = 6).

To improve the PK profile of the candidate compounds, efforts were focused on reducing their distribution volume to shorten the t_1/2_.^46^ As mentioned earlier, lipophilicity improves absorption, brain penetration, and affinity for orexin receptors, while it increases distribution volume and prolongs t_1/2_. Alternatively, increasing protein binding can limit tissue transfer and reduce distribution volume, but it reduces CNS transfer and efficacy. The small distribution volume and moderate protein binding rate of vornorexant were achieved by adopting a 1,3-oxazinane motif, which balances reduced lipid solubility and in vitro activities at orexin receptors. The apparent distribution volume is 28.9 L in humans, equivalent to the total fluid volume,^47^ and is the smallest among the DORAs on the market (Table 2). After the radiolabeled drug was administered to animals, the tissue concentrations were not high, the radioactivity disappeared in most tissues within 24 hours after administration, and the radioactivity was excreted in the urine and feces.^46^ Consistent with these preclinical results, the plasma drug concentration reached C_max_ in 0.5–1.5 hours after a single oral administration to healthy volunteers and then decreased with a t_1/2_ of 1.32–3.25 hours.^47^ The shortest t_1/2_ among DORAs (approximately 2 hours) was achieved in humans (Figure 4). The protein binding rate of vornorexant is 94.5%–96.3%, which is higher than that of lemborexant (87.4%–88.7%)^48^ and lower than that of suvorexant and daridorexant (>99%).^37^^,^^49^ As it is generally believed that the unbound fraction in the plasma migrates to the CSF and reaches the CNS, these differences may reflect differences in their respective dosages, with lemborexant and vornorexant requiring a lower dose (5 mg) compared with suvorexant (20 mg) and daridorexant (50 mg).

**Table 2:** Pharmacokinetic parameters of DORAs.

Drug	Dose for pharmacokinetic study (mg/day)	C_max_ (ng/mL)	t_max_ (h)	AUC_0-∞_ (ng・h/mL)	t_1/2_ (h)	V_d_/F (L)	CL/F (L/h)	Reference
Suvorexant	20	291^a^	1.0	4290^a^	12.5^b^	57.1^a^^,^^c^	2.92^a^^,^^d^	MSD 2014^49^
Lemborexant	10	46.5	1.0	231^e^	16.7	2120	32.8	Landry et al., 2021^50^; Ueno et al., 2021^48^
Daridorexant	50	1230^f^	2.0	7430^f^	5.9^f^	31^c^^,^^f^	5.0^d^^,^^f^	Muehlan et al., 2018^51^
Vornorexant	10	289	0.750	1010	1.96	28.9	10.5	Kambe et al., 2023^47^

Pharmacokinetic data were collected after administration of a single dose to healthy adult volunteers.

Data are expressed as means except for t_max_, which is expressed as median.

a least squares mean

b harmonic mean

c volume of distribution at steady state

d total clearance

e AUC_0-24h_

f geometric mean

Abbreviations: AUC_0-∞_, area under the plasma concentration–time curve from time zero to infinity; AUC_0-24h_, AUC from zero to 24 h; CL/F, apparent clearance; C_max_, maximum plasma concentration; DORA, dual orexin receptor antagonist; t_max_, time to reach C_max_; t_1/2_, terminal half-life; V_d_/F, apparent volume of distribution.

The metabolites of vornorexant, M1 and M3 have shown antagonist activity at OX_1_ and OX_2_ receptors, although their activity is weaker than that of the unchanged drug. To clarify whether these active metabolites contribute to the drug’s efficacy, the penetration of vornorexant and its active metabolites into CSF, which is considered to approximate the unbound fraction in the brain, was investigated in rats.^46^ In the rat plasma, the relative abundance of analytes was M1 > unchanged drug > M3, whereas in the rat CSF, the order was unchanged drug > M1 > M3. At 1 hour after administration, the unchanged drug concentration in CSF reached a maximum concentration and was 11- and 5.9-fold higher than its in vitro effective concentrations (K_b_ value) for OX_1_ and OX_2_ receptors, respectively. The CSF concentrations of M1 and M3 also reached a maximum at 1 hour after administration. These concentrations were 2.3- and 1.1-fold (M1), and 1.5- and 0.9-fold (M3) higher than the respective in vitro effective concentrations, but remained lower than those of the unchanged drug. These results suggest that the unchanged drug mainly contributes to the pharmacological effects in rats. In contrast, the unchanged drug was the main component in the plasma in dogs (unchanged drug > M1 > M3)^46^ and humans (unchanged drug > M3 > M1).^47^ Assuming that there are no species-specific differences in brain penetration, this suggests that the unchanged form is the major component of CSF in dogs and humans and that it is primarily responsible for the pharmacological effect of vornorexant.^46^

In summary, vornorexant is rapidly absorbed and has a shorter t_1/2_ than other DORAs, similar to that of zolpidem, an ultra-short-acting hypnotic with a t_1/2_ of 1.78–2.30 hours (Figure 4). Notably, in rats, the t_1/2_ of receptor occupancy at OX_1_ and OX_2_ receptors for vornorexant is consistent with that in plasma drug concentration.^35^ Therefore, rapid elimination of vornorexant from the plasma suggests that vornorexant may reduce the risk of next-day residual effects. This assumption is underpinned by results of the pharmacodynamic assessment of vornorexant in healthy subjects, where only subtle and inconsistent differences were noted between vornorexant and placebo in several endpoints (Karolinska Sleepiness Scale (KSS), Digit Symbol Substitution Test (DSST), and psychomotor vigilance task) at 9 hours after dosing, whereas more potent and clearer effects were observed at earlier time points (1 and 4 hours after dose).^47^

## SLEEP-PROMOTING EFFECTS AND SAFETY OF VORNOREXANT IN PATIENTS WITH INSOMNIA

In a phase 2 study, the efficacy and safety of a single oral dose of vornorexant were investigated in 24 patients diagnosed with insomnia according to the Diagnostic and Statistical Manual of Mental Disorders, Fifth Edition, using a randomized, double-blind, crossover design. All patients received placebo or vornorexant at 5, 10, or 30 mg, and efficacy, including the primary endpoints of LPS and WASO by overnight PSG, and safety were evaluated.^52^

**Figure 5:**
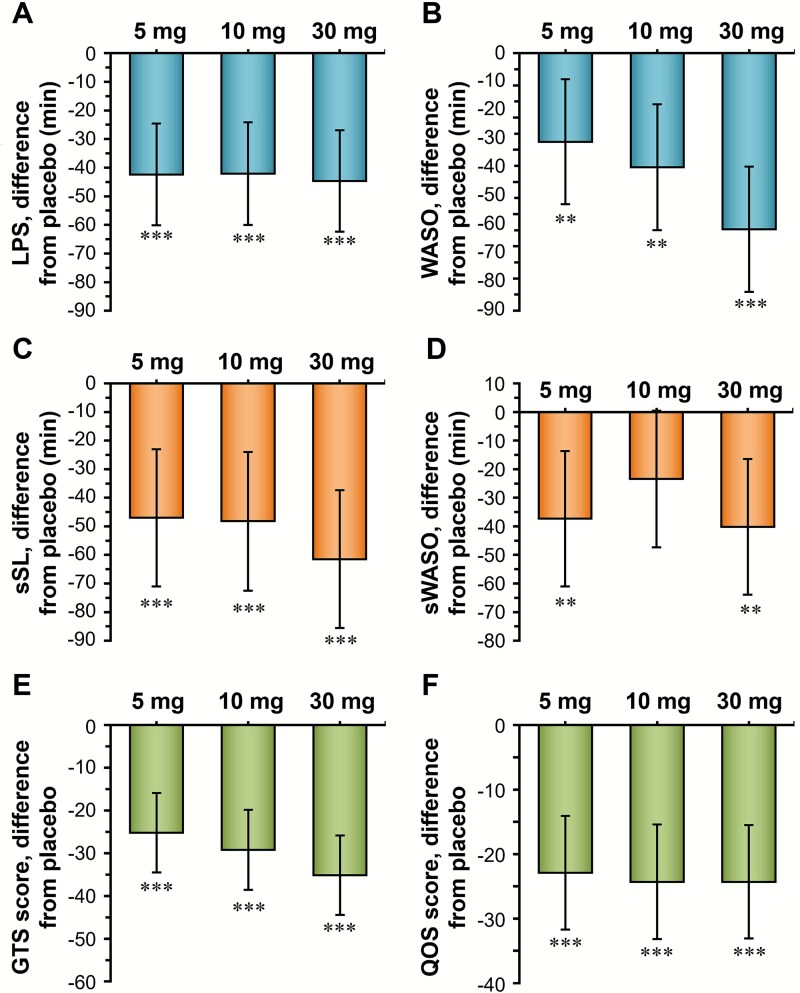
Effects of vornorexant on sleep in patients with insomnia (adapted from Uchiyama et al., 2022^52^). LPS (A) and WASO (B) measured with PSG, sSL (C) and sWASO (D) recorded with the sleep diary, and GTS score (E) and QOS score (F) assessed with the Leeds sleep evaluation questionnaire when dosed with placebo or vornorexant (5, 10, or 30 mg) for 2 weeks. Data represent the difference in change in the second week versus the placebo group as least squares mean with 95% confidence intervals. ^**^P<.01 vs. placebo, ^***^P<.001 vs. placebo: All *P*-values are nominal (not adjusted for multiplicity). GTS, getting to sleep; LPS, latency to persistent sleep; PSG, polysomnography; QOS, quality of sleep; s, subjective; SL, sleep latency; WASO, wake time after sleep onset.

Although the sample size of this study was small, the effects of vornorexant on sleep onset and suppression of awakening during the night were clearly demonstrated (Figure 5). With respect to the sleep onset effect, vornorexant shortened the LPS from 73 minutes (range 20–250 minutes) before administration to approximately 8–12 minutes. The least squares mean difference of LPS between the vornorexant group and the placebo group was −42.38 minutes, −42.10 minutes, and − 44.68 minutes for the 5-, 10-, and 30-mg doses, respectively (all *P* < .001, Figure 5A).^52^ The LPS of 8–12 minutes is within the definition of a “good sleeper.”^53^ With respect to the effect of suppressing awakening after sleep onset, vornorexant significantly shortened WASO, improving the least squares mean difference versus placebo by −27.52 (5 mg), −35.44 (10 mg), and − 54.69 (30 mg) minutes from 103 minutes at baseline (range 61–343 minutes) (all *P* <.01, Figure 5B).^52^ These hypnotic effects are consistent with the PK profile of vornorexant. Although LPS did not show a clear dose–response, the plasma concentration of vornorexant rose rapidly (Figure 4), suggesting that there is no difference in the time to reach an effective concentration at the doses of 5–30 mg. In contrast, the duration of the effective concentration of vornorexant increased with dose (Figure 4), suggesting that the effect on WASO strengthens with increasing dose. The PK profile of vornorexant appears to support rapid sleep onset and may help maintain activity through most of the night.

While PSG is an objective assessment using electroencephalography, subjective assessments reported by patients have also shown that vornorexant improves sleep. The sleep diary–based subjective sleep latency (sSL, all *P* < .001, Figure 5C) and subjective WASO (sWASO, *P* <.01 for 5 and 30 mg, Figure 5D) significantly improved with vornorexant compared with placebo. Notably, the Leeds Sleep Evaluation Questionnaire, a 10-item self-report scale designed to assess sleep quality, also showed significant improvements with vornorexant compared with placebo in terms of ease of getting to sleep (GTS, all *P* < .001, Figure 5E) and quality of sleep (QOS, all *P* <.001, Figure 5F).^52^

In the phase 2 study, vornorexant was well tolerated, and all adverse events were mild or moderate.^52^ The only adverse events that occurred in 2 or more patients were somnolence and nightmares at the 30-mg dose. Next-morning residual effects are often assessed using the KSS, DSST, and the occurrence of somnolence as reported by investigators and participants. In this study, vornorexant did not show pronounced score changes in the KSS or DSST compared with placebo.^52^ Therefore, vornorexant may have a low incidence of next-morning residual effects, at least within the dose range used in this study.

The results of this phase 2 study show that a single oral administration of vornorexant has sleep-inducing effects and is well tolerated. It should be noted that this phase 2 study is a small sample size trial (*n* = 24). Subsequently, the efficacy and safety of vornorexant have been confirmed in a phase 3 study; however, its clinical utility still requires further investigation in real-world clinical settings.

## CONCLUSIONS

Since their market introduction, DORAs have been widely used as effective hypnotics that are devoid of the shortcomings of conventional hypnotics, particularly the agents modulating GABA_A_ receptor signaling.^30–34^^,^^38^^,^^39^ Nonetheless, some doses of the hitherto used DORAs (suvorexant, lemborexant, and daridorexant)^49–51^ are associated with higher incidence of somnolence, suggesting the risk of next-day residual activities. Therefore, vornorexant, a DORA whose t_1/2_ is the shortest among DORAs in the market, equivalent to that of zolpidem, would be a useful option for those unsatisfied with currently available medications.

On the other hand, it is worth noting that to date, clear correlation has not been fully established between the PK profiles of DORAs and their efficacy or safety, and that multiple factors, including PK, brain dynamics and detailed pharmacological properties should be considered to discuss efficacy or safety associated with usage of DORAs. Indeed, a recent report using a random-effects model network meta-analysis showed that daridorexant outperformed lemborexant and suvorexant in terms of subjective total sleep time after 1 month, suggesting that daridorexant may more effectively improve sleep maintenance difficulties, despite having the shortest t_1/2_ among the three DORAs.^40^ Additionally, it has also been reported that lower dose but not higher dose of daridorexant increased the risk of somnolence.^40^ In contrast, daridorexant appears to have the lowest risk of somnolence among DORAs,^40^ which may be ascribed to a shorter t_1/2_ than lemborexant and suvorexant. Therefore, it is still premature to conclude that the efficacy of DORAs fully depends on their PK profiles, and the clinical utility of each DORA, including vornorexant, should ultimately be determined based on clinical results.

Although more clinical evidence is waited, addition of vornorexant can extend the utility of DORAs and eventually contribute to expanding the therapeutic options for insomnia.

## Data Availability

No new data were generated in support of this work.

## References

[1] Riemann D, Nissen C, Palagini L, Otte A, Perlis ML, Spiegelhalder K. The neurobiology, investigation, and treatment of chronic insomnia. *Lancet Neurol*. 2015;14:547–558. 10.1016/S1474-4422(15)00021-6 25895933

[2] Roth T . Insomnia: definition, prevalence, etiology, and consequences. *J Clin Sleep Med*. 2007;3:S7–S10. 10.5664/jcsm.26929 17824495 PMC1978319

[3] Kyle SD, Espie CA, Morgan K. "...Not just a minor thing, it is something major, which stops you from functioning daily": quality of life and daytime functioning in insomnia. *Behav Sleep Med*. 2010;8:123–140. 10.1080/15402002.2010.487450 20582756

[4] Sateia MJ, Buysse DJ, Krystal AD, Neubauer DN, Heald JL. Clinical practice guideline for the pharmacologic treatment of chronic insomnia in adults: an American academy of sleep medicine clinical practice guideline. *J Clin Sleep Med*. 2017;13:307–349. 10.5664/jcsm.6470 27998379 PMC5263087

[5] Atkin T, Comai S, Gobbi G. Drugs for insomnia beyond benzodiazepines: pharmacology, clinical applications, and discovery. *Pharmacol Rev*. 2018;70:197–245. 10.1124/pr.117.014381 29487083

[6] Gravielle MC . Activation-induced regulation of GABA_A_ receptors: is there a link with the molecular basis of benzodiazepine tolerance? *Pharmacol Res*. 2016;109:92–100. 10.1016/j.phrs.2015.12.030 26733466

[7] Hindmarch I . Cognitive toxicity of pharmacotherapeutic agents used in social anxiety disorder. *Int J Clin Pract*. 2009;63:1085–1094. 10.1111/j.1742-1241.2009.02085.x 19570125

[8] Gustavsen I, Bramness JG, Skurtveit S, Engeland A, Neutel I, Mørland J. Road traffic accident risk related to prescriptions of the hypnotics zopiclone, zolpidem, flunitrazepam and nitrazepam. *Sleep Med*. 2008;9:818–822. 10.1016/j.sleep.2007.11.011 18226959

[9] Capiau A, Huys L, van Poelgeest E, et al. Therapeutic dilemmas with benzodiazepines and Z-drugs: insomnia and anxiety disorders versus increased fall risk: a clinical review. *Eur Geriatr Med*. 2023;14:697–708. 10.1007/s41999-022-00731-4 36576689 PMC10447278

[10] Lader M . Benzodiazepine harm: how can it be reduced? *Br J Clin Pharmacol*. 2014;77:295–301. 10.1111/j.1365-2125.2012.04418.x 22882333 PMC4014015

[11] de Lecea L, Kilduff TS, Peyron C, et al. The hypocretins: hypothalamus-specific peptides with neuroexcitatory activity. *Proc Natl Acad Sci USA*. 1998;95:322–327. 10.1073/pnas.95.1.322 9419374 PMC18213

[12] Sakurai T, Amemiya A, Ishii M, et al. Orexins and orexin receptors: a family of hypothalamic neuropeptides and G protein-coupled receptors that regulate feeding behavior. *Cell.* 1998;92:573–585. 10.1016/S0092-8674(00)80949-6 9491897

[13] Mieda M, Hasegawa E, Kisanuki YY, Sinton CM, Yanagisawa M, Sakurai T. Differential roles of orexin receptor-1 and -2 in the regulation of non-REM and REM sleep. *J Neurosci*. 2011;31:6518–6526. 10.1523/JNEUROSCI.6506-10.2011 21525292 PMC3732784

[14] Chemelli RM, Willie JT, Sinton CM, et al. Narcolepsy in orexin knockout mice: molecular genetics of sleep regulation. *Cell.* 1999;98:437–451. 10.1016/S0092-8674(00)81973-X 10481909

[15] Hara J, Beuckmann CT, Nambu T, et al. Genetic ablation of orexin neurons in mice results in narcolepsy, hypophagia, and obesity. *Neuron.* 2001;30:345–354. 10.1016/S0896-6273(01)00293-8 11394998

[16] Aston-Jones G, Smith RJ, Moorman DE, Richardson KA. Role of lateral hypothalamic orexin neurons in reward processing and addiction. *Neuropharmacology.* 2009;56:112–121. 10.1016/j.neuropharm.2008.06.060 18655797 PMC2635332

[17] Grafe LA, Eacret D, Luz S, et al. Orexin 2 receptor regulation of the hypothalamic-pituitary-adrenal (HPA) response to acute and repeated stress. *Neuroscience.* 2017;348:313–323. 10.1016/j.neuroscience.2017.02.038 28257896 PMC6322837

[18] Lin L, Faraco J, Li R, et al. The sleep disorder canine narcolepsy is caused by a mutation in the hypocretin (orexin) receptor 2 gene. *Cell.* 1999;98:365–376. 10.1016/S0092-8674(00)81965-0 10458611

[19] Sakurai T . The neural circuit of orexin (hypocretin): maintaining sleep and wakefulness. *Nat Rev Neurosci*. 2007;8:171–181. 10.1038/nrn2092 17299454

[20] Bonaventure P, Shelton J, Yun S, et al. Characterization of JNJ-42847922, a selective orexin-2 receptor antagonist, as a clinical candidate for the treatment of insomnia. *J Pharmacol Exp Ther*. 2015;354:471–482. 10.1124/jpet.115.225466 26177655

[21] Morairty SR, Revel FG, Malherbe P, et al. Dual hypocretin receptor antagonism is more effective for sleep promotion than antagonism of either receptor alone. *PLoS One*. 2012;7:e39131. 10.1371/journal.pone.0039131 22768296 PMC3388080

[22] Brisbare-Roch C, Dingemanse J, Koberstein R, et al. Promotion of sleep by targeting the orexin system in rats, dogs and humans. *Nat Med*. 2007;13:150–155. 10.1038/nm1544 17259994

[23] Hoever P, Dorffner G, Beneš H, et al. Orexin receptor antagonism, a new sleep-enabling paradigm: a proof-of-concept clinical trial. *Clin Pharmacol Ther*. 2012;91:975–985. 10.1038/clpt.2011.370 22549286 PMC3370822

[24] Roth T, Black J, Cluydts R, et al. Dual orexin receptor antagonist, almorexant, in elderly patients with primary insomnia: a randomized, controlled study. *Sleep.* 2017;40:zsw034. 10.1093/sleep/zsw034 28364509

[25] Hoch M, Hoever P, Theodor R, Dingemanse J. Almorexant effects on CYP3A4 activity studied by its simultaneous and time-separated administration with simvastatin and atorvastatin. *Eur J Clin Pharmacol*. 2013;69:1235–1245. 10.1007/s00228-012-1470-8 23334403

[26] Beuckmann CT, Suzuki M, Ueno T, Nagaoka K, Arai T, Higashiyama H. In vitro and in silico characterization of lemborexant (E2006), a novel dual orexin receptor antagonist. *J Pharmacol Exp Ther*. 2017;362:287–295. 10.1124/jpet.117.241422 28559480

[27] Cox CD, Breslin MJ, Whitman DB, et al. Discovery of the dual orexin receptor antagonist [(7R)-4-(5-chloro-1,3-benzoxazol-2-yl)-7-methyl-1,4-diazepan-1-yl][5-methyl-2-(2H–1,2,3-triazol-2-yl)phenyl]methanone (MK-4305) for the treatment of insomnia. *J Med Chem*. 2010;53:5320–5332. 10.1021/jm100541c 20565075

[28] Roch C, Bergamini G, Steiner MA, Clozel M. Nonclinical pharmacology of daridorexant: a new dual orexin receptor antagonist for the treatment of insomnia. *Psychopharmacol (Berl)*. 2021; 238:2693–2708. 10.1007/s00213-021-05954-0 PMC8455402 34415378

[29] Brunner DP, Dijk DJ, Münch M, Borbély AA. Effect of zolpidem on sleep and sleep EEG spectra in healthy young men. *Psychopharmacol (Berl).* 1991;104:1–5. 10.1007/BF02244546 1881993

[30] Michelson D, Snyder E, Paradis E, et al. Safety and efficacy of suvorexant during 1-year treatment of insomnia with subsequent abrupt treatment discontinuation: a phase 3 randomised, double-blind, placebo-controlled trial. *Lancet Neurol*. 2014;13:461–471. 10.1016/S1474-4422(14)70053-5 24680372

[31] Mignot E, Mayleben D, Fietze I, et al. Safety and efficacy of daridorexant in patients with insomnia disorder: results from two multicentre, randomised, double-blind, placebo-controlled, phase 3 trials. *Lancet Neurol*. 2022;21:125–139. 10.1016/S1474-4422(21)00436-1 35065036

[32] Muehlan C, Vaillant C, Zenklusen I, Kraehenbuehl S, Dingemanse J. Clinical pharmacology, efficacy, and safety of orexin receptor antagonists for the treatment of insomnia disorders. *Expert Opin Drug Metab Toxicol*. 2020;16:1063–1078. 10.1080/17425255.2020.1817380 32901578

[33] Takaesu Y, Suzuki M, Moline M, et al. Effect of discontinuation of lemborexant following long-term treatment of insomnia disorder: secondary analysis of a randomized clinical trial. *Clin Transl Sci*. 2023;16:581–592. 10.1111/cts.13470 36564964 PMC10087073

[34] Yardley J, Kärppä M, Inoue Y, et al. Long-term effectiveness and safety of lemborexant in adults with insomnia disorder: results from a phase 3 randomized clinical trial. *Sleep Med*. 2021;80:333–342. 10.1016/j.sleep.2021.01.048 33636648

[35] Hikichi H, Tokumaru Y, Taruta A, et al. Preclinical pharmacological profiles of vornorexant, a novel potent dual orexin receptor antagonist. *J Pharmacol Exp Ther*. 2025;392:103624. 10.1016/j.jpet.2025.103624 40570549

[36] Rappas M, Ali AAE, Bennett KA, et al. Comparison of orexin 1 and orexin 2 ligand binding modes using X-ray crystallography and computational analysis. *J Med Chem*. 2020;63:1528–1543. 10.1021/acs.jmedchem.9b01787 31860301 PMC7050010

[37] Treiber A, de Kanter R, Roch C, et al. The use of physiology-based pharmacokinetic and pharmacodynamic modeling in the discovery of the dual orexin receptor antagonist ACT-541468. *J Pharmacol Exp Ther*. 2017;362:489–503. 10.1124/jpet.117.241596 28663311

[38] Álamo C, Sáiz Ruiz J, Zaragozá AC. Orexinergic receptor antagonists as a new therapeutic target to overcome limitations of current pharmacological treatment of insomnia disorder. *Actas Esp Psiquiatr*. 2024;52:172–182. 10.62641/aep.v52i2.1659 38622003 PMC11015820

[39] Mogavero MP, Silvani A, Lanza G, DelRosso LM, Ferini-Strambi L, Ferri R. Targeting orexin receptors for the treatment of insomnia: from physiological mechanisms to current clinical evidence and recommendations. *Nat Sci Sleep*. 2023;15:17–38. 10.2147/NSS.S201994 36713640 PMC9879039

[40] Kishi T, Ikuta T, Citrome L, et al. Comparative efficacy and safety of daridorexant, lemborexant, and suvorexant for insomnia: a systematic review and network meta-analysis. *Transl Psychiatry*. 2025;15:211. 40555730 10.1038/s41398-025-03439-8 PMC12187915

[41] Mesens S, Krystal AD, Melkote R, et al. Efficacy and safety of seltorexant in insomnia disorder: a randomized clinical trial. *JAMA Psychiatry*. 2025;82:967–976. 10.1001/jamapsychiatry.2025.1999 PMC12351464 40802194

[42] Suzuki R, Nozawa D, Futamura A, et al. Discovery and in vitro and in vivo profiles of N-ethyl-N-[2-[3-(5-fluoro-2-pyridinyl)-1H-pyrazol-1-yl]ethyl]-2-(2H-1,2,3-triazol-2-yl)-benzamide as a novel class of dual orexin receptor antagonist. *Bioorg Med Chem*. 2015;23:1260–1275. 10.1016/j.bmc.2015.01.044 25693785

[43] Obach RS, Lombardo F, Waters NJ. Trend analysis of a database of intravenous pharmacokinetic parameters in humans for 670 drug compounds. *Drug Metab Dispos*. 2008;36:1385–1405. 10.1124/dmd.108.020479 18426954

[44] Smith DA, Beaumont K, Maurer TS, Di L. Volume of distribution in drug design. *J Med Chem*. 2015;58:5691–5698. 10.1021/acs.jmedchem.5b00201 25799158

[45] Futamura A, Suzuki R, Tamura Y, et al. Discovery of ORN0829, a potent dual orexin 1/2 receptor antagonist for the treatment of insomnia. *Bioorg Med Chem*. 2020;28:115489. 10.1016/j.bmc.2020.115489 32482533

[46] Konno Y, Kamigaso S, Toki H, et al. Preclinical metabolism and the disposition of vornorexant/TS-142, a novel dual orexin 1/2 receptor antagonist for the treatment of insomnia. *Pharmacol Res Perspect*. 2024;12:e1183. 10.1002/prp2.1183 38491717 PMC10943176

[47] Kambe D, Hasegawa S, Imadera Y, et al. Pharmacokinetics, pharmacodynamics and safety profile of the dual orexin receptor antagonist vornorexant/TS-142 in healthy Japanese participants following single/multiple dosing: randomized, double-blind, placebo-controlled phase-1 studies. *Basic Clin Pharmacol Toxicol*. 2023;133:576–591. 10.1111/bcpt.13930 37563858

[48] Ueno T, Ishida T, Aluri J, et al. Disposition and metabolism of [^14^C]lemborexant in healthy human subjects and characterization of its circulating metabolites. *Drug Metab Dispos*. 2021;49:31–38. 10.1124/dmd.120.000229 33144331

[49] MSD inc . Suvorexant common technicaldocument. 2014. https://www.pmda.go.jp/drugs/2014/P201400117/index.html. Japanese; Accessed June 24, 2025

[50] Landry I, Nakai K, Ferry J, et al. Pharmacokinetics, pharmacodyna,mics, and safety of the dual orexin receptor antagonist lemborexant: findings from single-dose and multiple-ascending-dose phase 1 studies in healthy adults. *Clin Pharmacol Drug Dev*. 2021;10:153–165. 10.1002/cpdd.817 32468649 PMC7891412

[51] Muehlan C, Heuberger J, Juif PE, Croft M, van Gerven J, Dingemanse J. Accelerated development of the dual orexin receptor antagonist ACT-541468: integration of a microtracer in a first-in-human study. *Clin Pharmacol Ther*. 2018;104:1022–1029. 10.1002/cpt.1046 29446069

[52] Uchiyama M, Kambe D, Imadera Y, Kajiyama Y, Ogo H, Uchimura N. Effects of TS-142, a novel dual orexin receptor antagonist, on sleep in patients with insomnia: a randomized, double-blind, placebo-controlled phase 2 study. *Psychopharmacol (Berl)*. 2022;239:2143–2154. 10.1007/s00213-022-06089-6 PMC9205809 35296912

[53] Hertenstein E, Gabryelska A, Spiegelhalder K, et al. Reference data for polysomnography-measured and subjective sleep in healthy adults. *J Clin Sleep Med*. 2018;14:523–532. 10.5664/jcsm.7036 29609718 PMC5886429

